# Dissecting *in silico* Mutation Prediction of Variants in African Genomes: Challenges and Perspectives

**DOI:** 10.3389/fgene.2019.00601

**Published:** 2019-06-25

**Authors:** Christian Domilongo Bope, Emile R. Chimusa, Victoria Nembaware, Gaston K. Mazandu, Jantina de Vries, Ambroise Wonkam

**Affiliations:** ^1^Department of Pathology, Faculty of Health Sciences, University of Cape Town, Cape Town, South Africa; ^2^Departments of Mathematics and Computer Sciences, Faculty of Sciences, University of Kinshasa, Kinshasa, Democratic Republic of Congo; ^3^Department of Medicine, Faculty of Health Sciences, University of Cape Town, Cape Town, South Africa; ^4^Institute of Infectious Diseases and Molecular Medicine, Faculty of Health Sciences, University of Cape Town, Cape Town, South Africa

**Keywords:** African genome, incidental findings, actionable variants, whole exome sequencing, whole genome sequencing, precision medicine, pathogenicity

## Abstract

Genomic medicine is set to drastically improve clinical care globally due to high throughput technologies which enable speedy *in silico* detection and analysis of clinically relevant mutations. However, the variability in the *in silico* prediction methods and categorization of functionally relevant genetic variants can pose specific challenges in some populations. *In silico* mutation prediction tools could lead to high rates of false positive/negative results, particularly in African genomes that harbor the highest genetic diversity and that are disproportionately underrepresented in public databases and reference panels. These issues are particularly relevant with the recent increase in initiatives, such as the Human Heredity and Health (H3Africa), that are generating huge amounts of genomic sequence data in the absence of policies to guide genomic researchers to return results of variants in so-called actionable genes to research participants. This report (i) provides an inventory of publicly available Whole Exome/Genome data from Africa which could help improve reference panels and explore the frequency of pathogenic variants in actionable genes and related challenges, (ii) reviews available *in silico* prediction mutation tools and the criteria for categorization of pathogenicity of novel variants, and (iii) proposes recommendations for analyzing pathogenic variants in African genomes for their use in research and clinical practice. In conclusion, this work proposes criteria to define mutation pathogenicity and actionability in human genetic research and clinical practice in Africa and recommends setting up an African expert panel to oversee the proposed criteria.

## Introduction

High throughput technologies in “omics” research are expected to improve clinical care globally through genomic medicine. However, the categorization and criteria to infer variants’ pathogenicity differs around the world and can pose specific challenges in some populations (Dorschner et al., 2013; Green et al., 2013; MacArthur et al., 2014; Amendola et al., 2015; Hunter et al., 2016; Ichikawa et al., 2017; Kwak et al., 2017; Lacaze et al., 2017; Tang et al., 2018). Particularly, in African genomes that harbor the highest genetic diversity, it is possible that most *in silico* prediction tools could lead to the highest rate of false positive/negative results (Martin et al., 2018). The H3Africa Consortium has significantly contributed to reducing the dearth of genomic research on the African continent by supporting African genomics researchers and developing policies (Dandara et al., 2014; H3Africa, 2017). However, in the current genomics landscape, it is particularly challenging to interpret some variants found in African genomes, i.e., to determine whether that variant is common or rare, benign or pathogenic. Firstly, approaches to determine the rareness of a variant are based on exploring publicly available genome reference databases in which African data are under-represented (Lek et al., 2016; Popejoy and Fullerton, 2016). In addition, most of the current well-established bioinformatics tools, variant calling pipelines, are benchmarked using non-African populations and most of the variants deposited in the public database are from non-African populations (Pabinger et al., 2012; Bao et al., 2014). Secondly, the high genetic diversity of African populations means that genomic studies are likely to detect many novel variants that are yet to be described in current public databases (Lebeko et al., 2017). Thirdly, there is a lack of evidence-based policies and guidelines to inform the characterization of actionable genes in African genomic research. A guideline on feeding back findings was recently developed by H3Africa; while this is a commendable achievement, it lacks the support of published empirical evidence^1^. This latter point is particularly important given the recent call from the American College of Medical Genetics (ACMG) to investigate pathogenic variants in so-called actionable genes that could potentially have direct clinical benefit, and to return the results to research participants (ACMG, 2013). This will open up a series of ethically relevant questions (Kiezun et al., 2012; MacArthur et al., 2014; Parker and Kwiatkowski, 2016), such as the definition of actionability and relevance to personalized medicine in a context of often scarce human and material resources, and ill-equipped healthcare systems (Masimirembwa et al., 2014).

To address these multiple challenges, and particularly that of variant interpretation in African genomes, it is appropriate to develop new pipelines using African genetics data or to benchmark existing bioinformatics pipeline tools using African populations to account for African genetic diversity. This paper aims to (i) provide an inventory of existing Whole Exome/Genome data from Africans that could help develop an African reference genome build, improve reference panels, and explore the frequency of pathogenic variants in actionable genes and related challenges; (ii) review available *in silico* prediction mutation tools and criteria for categorization of pathogenicity of novel variants; and (iii) propose recommendations for analyzing pathogenic variants in African genomes for their use in research and clinical practice.

## Current Challenges of WES/WGS Data Interpretation in Africans

Mastering of genome sequencing pipelines and downstream analysis are important for inferring meaningful information, such as detection of variants in medically relevant genes, from high throughput data such as Next Generation Sequencing (NGS), Whole Exome Sequencing (WES), or Whole Genome Sequencing (WGS). However, data processing, deep sequencing, and meticulous downstream analysis of WES/WGS still constitute a challenge in most of the current pipelines and tools. In addition, there are still some challenges, such as the interpretation of rare missense variants, reliability, and accuracy of pipelines for sequence alignments, variant calling, and data analysis, for the WES and WGS data of African populations (Wang et al., 2013; Rabbani et al., 2014; Bertier et al., 2016; Popejoy and Fullerton, 2016). To address some of these challenges, a plethora of bioinformatics algorithms and pipelines have been developed (Pabinger et al., 2012; Hentzsche et al., 2016; Xu, 2018). Current practice is to use existing variant calling pipelines, but this raises a number of questions, including how are universally reliable and accurate current WES/WGS bioinformatics tools and pipelines benchmarked using non-African data? What is the true proportion of African population data in the current reference genome builds that are publicly available, taking into account the variable level of admixture of African Americans who tend to be considered proxies of Africans in these databases [the Genome Reference Consortium Human Genome (GRCh3) and University of California, Santa Cruz (UCSC)] (Kuhn et al., 2009; Fujita et al., 2011; Leipzig, 2017)? Addressing these challenges will require that genomic research communities from the African continent develop an African benchmark bioinformatics pipeline to analyze genomic data that includes genetic diversity found in the African populations, and engage in a major effort in constructing an African-specific reference panel.

African populations in current reference panels are not representative of more differentiated population groups within Africa. Variant calling from NGS data is based on alignment to a single reference genome, which is problematic for diverse regions or populations, such as African populations. There is great opportunity in improving read alignment and variant calling for African genomes. A genome reference graph for alignment and variant calling may capture natural variation among populations, particularly populations of high diversity with low level of linkage disequilibrium.

Repetitive DNA sequences are abundant in a broad range of species, from bacteria to mammals, and they cover nearly half of the human genome. The other main issue is that repeats have always presented technical challenges for sequence alignment and assembly programs. NGS projects, with their short read lengths and high data volumes, have made these challenges more difficult. From a computational perspective, repeats create ambiguities in alignment and assembly, which, in turn, can produce biases and errors when interpreting results. Simply ignoring repeats is not an option, as this creates problems of its own and may mean that important biological phenomena are missed. Variation in repeats can alter the expression of genes, and changes in the number of repeats have been linked to certain human diseases. Unfortunately, the molecular characterization of these repeats has been hampered by technical limitations related to cloning, sequencing techniques, and alignment algorithms (Dilthey et al., 2014; Marcus et al., 2014; Church et al., 2015; Paten et al., 2017).

Fortunately, the number of genomic researchers in Africa is on the rise, which has led to an increase in African genomic data and publications (The H3Africa Consortium, 2014; Uthman et al., 2015; Mulder et al., 2016; Ndiaye Diallo et al., 2017). The increase in African genomic research has the potential to narrow the research gap between Africa and the rest of the world and can also improve implementation of genomic medicine. Therefore, we propose to use the available data to (i) develop Bioinformatics tools using African data, particularly for populations from sub-Saharan Africa who have the highest genetic diversity and low levels of admixture with European or Asian populations; (ii) benchmark existing tools using available African population data; and (iii) there is an urgent need for a centralized repository of publicly available African genomic data with annotated variants based on their pathogenicity, in order to increase our understanding of continental genomic diversity (Jongeneel et al., 2017; Mulder et al., 2017; Ahmed et al., 2018). To help initiate such endeavors we have provided here an inventory of African Whole Exome and Whole Genome data that are currently available to our knowledge (Table 1).

**Table 1:** Published whole exome and genomes data from sub-Saharan Africa.

Country	Region	Individuals	References
**Exomes**			
Botswana	Southern Africa	164	Retshabile et al., 2018
Uganda	Southern Africa	150	Retshabile et al., 2018
Ghanaian	Western Africa	1032	Kodaman et al., 2017
Tunisia	North Africa	7 19	Hamdi et al., 2018 Ben Rekaya et al., 2018
Morocco	North Africa	3	Bousfiha et al., 2017
Cameroonian	Central Africa	179	Sickle Cell Disease Project (Unpublished)
Congo	Central Africa	23	Sickle Cell Disease Project (Unpublished)
Black Xhosa (SA)	Southern Africa	25	Hearing Impairment Project (Unpublished)
African Caribbean (ACB)	Caribbean	98	1000 Genomes project
Esan in Nigeria (ESN)	Western Africa	111	1000 Genomes project
Mende in Sierra Leone (MSL)	Western Africa	98	1000 Genomes project
Yoruba in Ibadan, Nigeria (YRI)	Western Africa	100	1000 Genomes project
Luhya in Webuye, Kenya (LWK)	East Africa	106	1000 Genomes project
African Ancestry in Southwest USA (ASW)	North America	68	1000 Genomes project
Gambia in Western Division, The Gambia (GWD)	Western Africa	120	1000 Genomes project
African American	North America	761	Auer et al., 2012
**Genomes**			
Baganda, Banyarwanda, Barundi (Uganda); Luhya, Kikuyu, Kalenjin (Kenya); Sotho, Zulu (South Africa); Yoruba, Igbo (Nigeria); Ga-Adangbe (Ghana); Jola, Fula, Wolof, Mandika (Gambia); Amhara, Oromo, Somali (Ethiopia)	Sub Sahara (Eastern Africa, Southern Africa, Western Africa, Southern Africa)	320	Gurdasani et al., 2015
Kombo (Gambia)	Western Africa	2560	Jallow et al., 2009
South Africa	Southern Africa	13	Kramvis et al., 2002
Ovamboland (Namibia) Angola Madagascar	Southern Africa Southern Africa Madagascar	23 2 1	Kramvis et al., 2005
Algeria, Morocco, Libya, Tunisia, Tuareg Congo, Gabon, Cameroun, Nigeria	Northern Africa Sub Sahara	25 59	Fadhlaoui-Zid et al., 2013
Uganda Zimbabwean	Sub Sahara	112 174	Venner et al., 2016
Mandinka II, Serehule, Bambara, Malike, FulaII, FulaI, MandikaI, Wollof, Serere, Manjogo, Jola Mossi, Kasem, Yoruba, Namkam, Semi-Bantu, Akans, Bantu Kauma, Chonyi, WabondeI, Kambe, Luhya, Maasai, Wasambaa, Giriama, Mzigua Ari, Anuak, Sudanese, Gumuz Oromo, Somali, Wolayta, Afar, Tigray, Amhara Nama, Karretjie, Khomani, Malawi, Herero, Khwe, Ixu, HU/’Hoansi, Amaxhosa Sebantu	West African Niger-Congo Central West African Niger-Congo East Africa Niger-Congo East Africa Nilo-Saharan East Africa Afroasiatic Khoesan	2504	Busby et al., 2016
African American	African American	35370	Ng et al., 2014
Ethiopian (Weth)	Sub Sahara	120	Tekola-Ayele et al., 2015
South Africa	Southern Africa	24 (8 Colored, 16 Black)	Choudhury et al., 2017


## *In Silico* Prediction of Mutations and Challenges

The accuracy of variant calling pipelines (Li et al., 2009; DePristo et al., 2011; Wei et al., 2011; Garrison and Marth, 2012; Koboldt et al., 2012; Wilm et al., 2012; Lai et al., 2016) is a major step prior to the downstream *in silico* prediction of mutations. Nevertheless, a challenge remains in downstream NGS variant calling analysis, i.e., to distinguish pathogenic mutations and rare non-pathogenic variants from most of the annotating variant calling pipelines. The accuracy of *in silico* prediction of rare and actionable disease-causing genetic variants for the detection of pathogenic rare mutations and polymorphisms is the greatest challenge. Variant calling pipelines generate large numbers variations erroneously, which may contain rare, common genetic variants, false positives, and false negatives (Dong et al., 2015). Further downstream analysis such as variant annotations, variant filtrations, and prioritization methods are conducted to annotate variant genomic features, gene symbols, exonic functions, and amino acid modifications (Bao et al., 2014). Different *in silico* prediction algorithms are implemented to annotate disease-causing mutations based on the following information from the variants: (i) sequence homology (Reva et al., 2011), (ii) protein structure (Ng and Henikoff, 2006; Teng et al., 2009), (iii) evolutionary conservation (Cooper et al., 2010), (iv) the frequency of pathogenicity (Kobayashi et al., 2017), and (v) change in ancestry. Most of the *in silico* prediction methods interact with public databases to incorporate updated variant information in order to enhance annotation prediction efficiency. The incorporated information is mainly the minor allele frequency (MAF), experimental clinical assay information and deleterious prediction of variants (Pabinger et al., 2012). The majority of *in silico* prediction tools provide a reduced number of annotations from large background errors of detected variants. To annotate, filter, and prioritize accurately variant calling, researchers developed pipelines combined with different annotation tools and databases. Germline and somatic mutation databases, such as ANNOVAR (Wang et al., 2010; Yang and Wang, 2015), Human Gene Mutation Database^2^, dbSNP^3^ (Sherry et al., 2001), and GENEKEEPER^4^ and others are important for evaluating variants. Liu et al. (2011) developed a robust database called dbNSFP, which combines the prediction scores of six prediction algorithms namely SIFT (Kumar et al., 2009), PolyPhen-2 (Adzhubei et al., 2010), LRT (Chun and Fay, 2009), MutationTaster (Schwarz et al., 2010), Mutation Assessor (Reva et al., 2011), FATHMM (Chun and Fay, 2009; Shihab et al., 2013), and conservative score tools namely GERP++ (Davydov et al., 2010), SiPhy (Garber et al., 2009), and PhyloP (Doerks et al., 2002) and then compiles the scores of these tools into one (Liu et al., 2011). ClinVar is a commonly used database for germline variants, namely pathogenic and benign and provides related clinical and experimental information^5^ (Landrum et al., 2016).

After annotation, it is recommended to filter annotated variants from many tools using two approaches (i) free hypothesis, to cast the vote of the annotated variant filters for “Deleterious or damaging disease-causing (D)” or “disease-causing automatic (A)” among annotation prediction tools based on a defined cut-off (∼50%); and (ii) non-free hypothesis, which provides a list of known genes of the studies with another level of prediction cut-off (∼25%). The cut-off for both hypotheses is study related.

*In silico* prediction of mutations in the context of African populations introduces additional specific challenges that are partly related to the use of non-African populations to benchmark *in silico* prediction pipelines and the low proportion of African population data in most of the interrogated databases. Another challenge when working with African population data is the annotation of common variants specific to African populations, which can be considered as pathogenic variants when using public databases. This emphasizes the need for a guideline, which defines approaches to infer pathogenicity variants in African populations.

### Predicting Pathogenic Variants and Challenges

In the literature and in most annotation databases, the classification of pathogenicity differs (Sherry et al., 2001; Wang et al., 2010; Yang and Wang, 2015; Landrum et al., 2016; McLaren et al., 2016). Nevertheless, a common strategy to define pathogenicity involves combining results from many annotation pipelines (Lebeko et al., 2017). Further downstream analyses are gene network analysis and gene enrichment. The purpose of these analyses is to investigate the level of interactions between genes and the annotated variants associated with human phenotypes and then mine affected biological processes, networks, pathways, and molecular functions (Bindea et al., 2009; Warde-Farley et al., 2010; Lebeko et al., 2017).

In the comprehensive standards and guidelines, ACMG and the Association for Molecular Pathology (AMP) define the nomenclature for variants (Table 2). Recommendations for laboratories and clinicians to return incidental findings (IFs) has led to interest toward defining criteria and mechanisms for evaluating pathogenicity and the frequencies of IFs in different populations. For example, Dorschner et al. (2013) analyzed actionable pathogenic variants in 500 European and 500 participants of African descent using exome data. The classifications for pathogenicity (Table 2) included allele frequency of the variants, segregation evidence, and the number of the patients affected with the variants and their status as a *de novo* mutation. The results showed major discrepancies in the frequencies of pathogenic variants among Europeans versus Africans, with an estimated frequency of ∼3.4% for those of European descent and ∼1.2% for those of African descent. In a similar study, Amendola et al. (2015) investigated IFs in 6503 with 4300 Europeans and 2203 individuals of African descent. In addition, functionally disruptive variant categories were added which represent the expected pathogenic variants as truncating and misplace-causing variants. To validate the results, a comparative analysis was conducted with other clinical and research genetic laboratories and *in silico* pathogenicity scores. The results also showed that those of African descent had a scientifically lower proportion (nearly 50%) of a pathogenic variant in actionable genes compared to European participants. This lower proportion found in both studies could be due to the underrepresentation of populations of African descent in the literature and publicly available databases.

**Table 2:** Variants pathogenicity categorization.

References	Categorization	Interpretation
Dorschner et al., 2013	Pathogenic	Allele frequency of the identified variant is below cutoff AND segregation can be found in at least two unrelated families
	Likely pathogenic variant of uncertain significance (VUS)	Allele frequency of the identified variant is below cutoff AND identified in at least three unrelated individuals
	VUS	Allele frequency of the discovered variant is below cutoff AND present in less than three unrelated affected individuals
	Likely benign VUS	Allele frequency of the identified variant is below cutoff AND/OR seen in combination with a known pathogenic mutation
Richards et al., 2015 ACGM and AMP	Pathogenic very strong	Null variant (non-sense, frameshift, initiation codon, single or multiexon deletion) in a gene is a known mechanism of disease
	Pathogenic strong	Similar amino acid modification which was previously considered as pathogenic variant independent of nucleotide change
	Pathogenic moderate	Localize in a mutational critical region and very important functional domain without benign variation
	Pathogenic supporting	Cosegregation with disease located in many affected family members in a gene well known to be the cause the disease
	Benign standalone	Allele frequency is greater than 5% in Exome Sequencing Project, 1000 Genomes Project, or Exome Aggregation Consortium
	Benign strong	Allele frequency is greater than expected for disorder
	Benign supporting	Missense variant in a gene for which primarily truncating variants are known to cause disease


Taking into account the high level of admixture of European ancestry among African Americans and the highest level of diversity among Africans, and poor representativity in public databases as well little clinical genetic research from Africa that is publicly available, it is likely that a similar study could even lead to a much lower proportion of IFs in sub-Saharan African populations. This indicates that there is an urgent need to improve criteria to categorize the pathogenicity when studying African populations, stressing for example investigating an appropriate number of ethnically matched control populations.

### Variants Actionability and Challenges

The Clinical Genome Resource (ClinGen) defines actionability as clinically prescribed interventions specific to the genetic disorder under consideration that is effective for prevention or delay of clinical disease, lowered clinical burden, or improved clinical outcomes in a previously undiagnosed adult and suggested a metric to score clinical actionability (Hunter et al., 2016). Interventions include patient management (e.g., risk-reducing surgery), surveillance, or specific circumstances the patients should avoid (e.g., certain types of anesthesia). The actionability includes interventions to improve outcomes for at-risk family members. Genetic testing recommendations for at-risk family members alone, however, were not considered sufficient to meet the criteria for actionability. In addition, actionability did not include reproductive decision-making.

Alternatively, the 100,000 Genomes Project protocol defines actionable genes as variants with a significant potential to prevent disease morbidity and mortality, if identified before symptoms become apparent. The variants with potentially severe impacts are clinically actionable causes of rare disease, where a healthcare intervention or screening programs might prevent an untoward outcome. The variants are known to result in illness or disability that is clinically significant, severely or moderately life threatening and clinically actionable. It should be emphasized that the exact criteria for considering whether a variant is considered actionable or not, and serious or not, is context-dependent and in some instances only emerges during the process of seeking ethical approval for the study (Genomics England, 2017).

The accepted process consists of defining actionability of the variant and a pathogenicity classification criterion. Both processes are evaluated, inspected and validated by a group of experts (Richards et al., 2015; Hunter et al., 2016). In the African context with highly genetically diverse populations, there is a need to update the proposed scoring metric to take into account the scarcity of health care professionals with medical genetics and genetic counseling skills, poorly equipped health facilities with a major disparity between urban and rural setting, and generally inadequate health systems.

## Return of Incidental Findings and Challenges in Africa

Next Generation Sequencing analysis could contribute to the improvement of patient care. This development has blurred the line between genomics and healthcare; the global recommendations on the identification and the return of IFs have raised some ethical concerns for genomic researchers, clinicians, and the public health authority. Prior to returning IFs, there is a need to have clear guidelines and recommendations on a list of potentially actionable genes and define how, what and when IFs should be returned (Ness, 2008; ACMG, 2013, 2015; Souzeau et al., 2016; Nowak et al., 2018). Wolf et al. (2008) published a paper, proposing a framework supporting disclosure of IFs to guide researchers particularly on informed consent, the handling process and the responsibility of institutional review boards. The process on informed consent regarding incidental findings returns is a separate ethical debate that will require appropriate consideration by various stakeholders through, for example, an African and international experts panel meeting with the aim to address (a) the definition of actionability in the context of Africa, (b) the priority list of conditions and related gene variants that are actionable in Africa, (c) the criteria for molecular validation of the variants found in genomic research for clinical use, (d) the clinical environment necessary for returning such results and by which category of health professionals, as most African settings do not have medical genetic services, and (e) the process of wording and integrating informed consent for incidental findings in genomic research in Africa. In the United States, the ACMG has provided a guideline and recommendations to evaluate the cost-effectiveness of returning pathogenic variants for 56 specific genes considered medically actionable (ACMG, 2013, 2015). In Europe, the EuroGenTest and the European Society of Human Genetics recently presented guidelines for diagnostic NGS, including a rating system for diagnostic tests (Matthijs et al., 2016). In the United Kingdom, the Association for Clinical genetic Science (ACGS) has also released a guideline for the evaluation of pathogenicity and reporting of sequence variants in clinical molecular genetics (Wallis et al., 2013). To the best of our knowledge, there are no evidence-based recommendations for African researchers and clinicians on how to report IFs (de Vries and Pepper, 2012; Sookrajh et al., 2015). This is not a surprise due to the fact that African populations and the diaspora are underrepresented in most of the genetics studies, which questions the universal applicability of the genetic findings in large genome studies, disease association and evolutionary genetic studies (Need and Goldstein, 2009; Rosenberg et al., 2010; Dorschner et al., 2013; Tiffin, 2014; Manrai et al., 2016).

Prior to proposing guidelines on the return of IFs for the African populations, researchers and clinicians should first conduct multiple genetics studies to characterize the nature of genes for both monogenic and complex diseases on multiple African populations. The results of such studies should first identify the frequency of pathogenic variants in actionable gene lists as defined, e.g., by the ACMG, annotate, and filter genes. An expert panel should validate the list of pathogenic and actionable variants, then conduct a comparative analysis with results from non-African populations (ACMG, 2013; Green et al., 2013; Kalia et al., 2017). The next step could be to define novel actionable genes and variants that are relevant to Africa, e.g., sickle cell disease or *APOL1* variants. Only after completing the aforementioned steps, African researchers and clinicians will be able to provide a comprehensive and clear guideline on which putative pathogenic genes may be returned. It should be noted that the framework on the return of IFs should covert different aspects such as ethical guidelines and genetic counseling. Due to the high diversity in the African population, the classification of pathogenic and actionable variants for the return of secondary findings is more challenging due to the following additional factors: (i) contextualizing the African definition of pathogenicity and actionable genes, (ii) the choice of control cohort for the validation among African populations (iii) the power of the sample size for the case and control cohort, and (iv) a list of actionable genes of the most prominent diseases in the African populations. These questions need to be considered and addressed prior to the development of African actionable gene standards and guideline for IFs. The guidelines and the list of African populations’ actionable genes to be returned as IFs is a major milestone toward personalized medicine.

## Conclusion and Perspectives

The power of high-throughput genomic technologies, particularly DNA sequencing, has potential to bridge the gap between genomic research and clinical care. However, this blurry line has opened several technical and ethical questions and concerns, especially in the context of African genomic research. With the highest genetic diversity found in individuals and communities across the African continent, the use of personalized medicine will be beneficial both to the continent and worldwide. The state of WES and WGS on the continent is in the early stages in terms of available genetic data, publications on genetic conditions, appropriately designed pipelines and bioinformatics tools. The process of handling IFs should be clearly discussed and defined by the African research community, clinicians, specifically on the categorization of the pathogenicity, and actionability of genes and variants in order to take advantage of the genomic technology.

We have provided a list of available WES and WGS data that can help in initiating, the development of bioinformatics pipelines suitable for African population genomic data, quantify the frequency of pathogenic and so-called actionable genes, and to develop appropriate policies for their investigation in genomic research. This requires African researchers and experts to be encouraged to share and make data available in public databases. This once again is an urgent call to set an African expert panel to categorize and refine criteria for pathogenicity and African actionability in human genetic research in Africa. We recommend that experts should prioritize the following steps: (1) define better criteria for classification of pathogenicity, and actionability, including relevant genes lists, that can be explored and return as IFs to research participant in Africa; (2) benchmark existing variant calling and *in silico* prediction pipelines for African genomic data or develop new pipelines using African data; (3) use hypothesis and non-hypothesis approaches *in silico* mutation prediction to avoid false positive mutation; (4) develop an African reference panel; and (5) Sanger sequencing to be done on the new variants for validation.

## Data Availability

All datasets analyzed for this study are cited in the manuscript and the Supplementary Files.

## Author Contributions

All authors contributed in conceiving, preparing, and revising the manuscript, approved the manuscript, and agreed to be accountable for all aspects of the presented work.

## Conflict of Interest Statement

The authors declare that the research was conducted in the absence of any commercial or financial relationships that could be construed as a potential conflict of interest.

## Footnotes

1 https://h3africa.org/wp-content/uploads/2018/05/H3Africa%20Feedback%20of%20Individual%20Genetic%20Results%20Policy.pdf

2 http://www.hgmd.cf.ac.uk/ac/index.php

3 https://www.ncbi.nlm.nih.gov/projects/SNP/

4 https://kewinc.com/analytics/

5 https://www.ncbi.nlm.nih.gov/clinvar/
